# Von Willebrand Factor Regulation in Patients with Acute and Chronic Cerebrovascular Disease: A Pilot, Case–Control Study

**DOI:** 10.1371/journal.pone.0099851

**Published:** 2014-06-17

**Authors:** Peter Kraft, Christiane Drechsler, Ignaz Gunreben, Bernhard Nieswandt, Guido Stoll, Peter Ulrich Heuschmann, Christoph Kleinschnitz

**Affiliations:** 1 Department of Neurology, University Hospital Würzburg, Würzburg, Germany; 2 Department of Internal Medicine, University Hospital Würzburg, Würzburg, Germany; 3 The Clinical Trial Center, University Hospital Würzburg, Würzburg, Germany; 4 Institute of Clinical Epidemiology and Biometry, Comprehensive Heart Failure Center, University of Würzburg, Würzburg, Germany; 5 Rudolf Virchow Center for Experimental Biomedicine, University of Würzburg, Würzburg, Germany; University of Münster, Germany

## Abstract

**Background and Purpose:**

In animal models, von Willebrand factor (VWF) is involved in thrombus formation and propagation of ischemic stroke. However, the pathophysiological relevance of this molecule in humans, and its potential use as a biomarker for the risk and severity of ischemic stroke remains unclear. This study had two aims: to identify predictors of altered VWF levels and to examine whether VWF levels differ between acute cerebrovascular events and chronic cerebrovascular disease (CCD).

**Methods:**

A case–control study was undertaken between 2010 and 2013 at our University clinic. In total, 116 patients with acute ischemic stroke (AIS) or transitory ischemic attack (TIA), 117 patients with CCD, and 104 healthy volunteers (HV) were included. Blood was taken at days 0, 1, and 3 in patients with AIS or TIA, and once in CCD patients and HV. VWF serum levels were measured and correlated with demographic and clinical parameters by multivariate linear regression and ANOVA.

**Results:**

Patients with CCD (158±46%) had significantly higher VWF levels than HV (113±36%, *P*<0.001), but lower levels than AIS/TIA patients (200±95%, *P*<0.001). Age, sex, and stroke severity influenced VWF levels (*P*<0.05).

**Conclusions:**

VWF levels differed across disease subtypes and patient characteristics. Our study confirms increased VWF levels as a risk factor for cerebrovascular disease and, moreover, suggests that it may represent a potential biomarker for stroke severity, warranting further investigation.

## Background

Plasmatic coagulation and platelets are critically involved in lesion development after ischemic stroke (IS) [1], [2]; however, their specific roles and their interplay with endothelial cells or other resident brain cells long remained elusive. Recently, the development of transgenic mouse models and specific antibodies has enabled the discovery of distinct pathways in pathological thrombus formation in animal models of IS [2], [3]. Von Willebrand factor (VWF) plays a key pathophysiologic role in arterial thrombus formation in the brain and heart [4], [5]. VWF is a large, multimeric glycoprotein that is exclusively synthesized in endothelial cells and megakaryocytes and circulates as multimers of varying size (up to 20 000 kDa). A disintegrin and metalloprotease with thrombospondin Type 1 repeats 13 (ADAMTS13) digests ultralarge VWF into smaller and less reactive molecules [4]. VWF mediates loose adhesion of platelets to the vascular wall and therefore, the earliest step of thrombus formation through binding to glycoprotein (Gp)Ib expressed on platelets [2], [6].

Research to date has shown that *in vivo* blockade of the GpIb–VWF axis resulted in the elimination of arterial thrombus formation in primates [7], restored vessel patency by dissolving platelet aggregates [8], and led to significantly reduced infarct volumes and better functional outcome in rodent stroke models [4]–[6] and larger animals [9]. Furthermore, targeting GpIb and its major ligand VWF has provided beneficial effects in IS models without a concomitant increase in bleeding complications, which led to the unique and intriguing concept of a potential “bleeding-free” antithrombotic approach [2]. Thus, targeting VWF-mediated platelet adhesion and activation is now considered as a potential target for stroke prevention and acute stroke treatment. However, a conservative view is warranted given that attempts to translate the findings with this approach from animal stroke studies to humans has yielded disappointing results [10].

Despite increasing evidence of a causal association between VWF levels and acute stroke risk in humans [11], [12] many questions remain unanswered. For instance, the processes involved in the regulation of VWF expression during ischemic stroke and the specific contribution of VWF to the preceding pathophysiologic events await clarification. Also, the relationship of VWF levels to other parameters, such as genetic polymorphisms [13] and demographic features [14], is not well understood. Additionally, only limited data exist on the regulation of VWF in patients with chronic cerebrovascular disease (CCD) [15]–[17].

The aim of the study was to identify demographic and clinical predictors of VWF serum levels and to evaluate whether VWF levels differ between acute cerebrovascular events and chronic cerebrovascular disease (CCD).

## Methods

### Data Collection

For addressing these objectives, a case-control study was performed. As cases, patients with acute (acute ischemic stroke [AIS], transitory ischemic attack [TIA]) and CCD were recruited. As controls, healthy volunteers (HV) from the local population were chosen. The participants were recruited at the Stroke Unit (in-patients diagnosed with TIA or AIS), at the out-patient clinic for CCD, or after a call for HV by means of posters at the Neurology Department, University Hospital of Würzburg, Germany, between September 2010 and January 2013. Inclusion criteria included blood withdrawal within 24 h after symptom onset in AIS (defined as acute ischemic lesion on brain imaging) and TIA (no acute lesion) patients, presentation with extra- and/or intracranial stenosis of the large cerebral arteries with (n = 66) or without (n = 51) history of AIS or TIA for the CCD group and no history of stroke, myocardial infarction, or peripheral arterial disease for the HV group. Exclusion criteria were hemorrhagic stroke, age<18 years, and known platelet dysfunction or plasmatic coagulation disorders based on a detailed medical history and collection of routine coagulation parameters. Overall, 116 patients with AIS or TIA, 117 patients with CCD, and 104 HV fulfilled the inclusion criteria and took part in the study.

In the patients with AIS or TIA, TOAST (Trial of Org 10172 in Acute Stroke Treatment) criteria [18] were applied in an adapted form: (1) cardioembolism; (2) large-artery atherosclerosis: (3) small-vessel occlusion; or (4) other determined or undetermined etiology. The National Institute of Health Stroke Scale (NIHSS) [19] and Barthel Index score [20] were calculated at patient admission. The latency between symptom onset and blood withdrawal, platelet inhibitor pretreatment, and modality of acute stroke therapy (thrombolysis vs. no thrombolysis) were registered.

### Blood Collection and Measurements

Blood was collected on day 0, 1, and 3 in the patients with acute cerebrovascular disease, and once in CCD patients and HV between 08.00 and 12.00 hours from an antecubital vein using a 21-gauge butterfly needle. Pre-analytic preparations for blood collection followed a specific standard operating procedure. Only non-hemolyzed blood samples were analysed. VWF, differential hematology parameters, and C-reactive protein (CRP) were analysed at the Division of Laboratory Medicine of the University Hospital Würzburg.

### Statistical Analysis

Continuous variables are expressed as mean with standard deviation or median with interquartile range, as appropriate. Categorical variables are expressed as percentages. The association between VWF concentrations and a range of demographic and clinical characteristics was explored: age, sex, neurological scales, disease modality (TIA or AIS), TOAST criteria, duration between symptom onset and blood withdrawal, NIHSS, Barthel Index, treatment modality (intravenous thrombolysis or not), and intake of platelet inhibitors in the days before blood withdrawal. *P* values for comparisons across groups of clinical and demographic characteristics were derived from analysis of variance (ANOVA), and the chi-square test, as appropriate. In order to identify potential predictors of VWF levels, a linear regression model was used that included all variables without co-linearity in a multivariate model adjusting for age and sex. Using this model, coefficients and the corresponding 95% confidence intervals (CIs) were estimated. VWF biomarker levels were compared between the different patients groups (AIS/TIA in-patients, CCD out-patients, or HV). Distribution was analyzed using the Kolmogorov–Smirnov test. Levels of VWF were assumed to take a normal distribution and were compared using ANOVA with a Bonferroni post-hoc test and additionally adjusted for age and sex. All reported *P* values are two-sided and a *P* value<0.05 was considered statistically significant. Analyses were performed using SPSS Version 21 and SAS software version 9.1 (SAS Institute Inc., Cary, NC).

### Ethics

Informed written consent was obtained from all participants. The study protocol was approved by the ethics committee of the medical faculty of the University of Würzburg, Germany (reference number 65/2010). Study participation had no influence on treatment and patient care.

## Results

### Descriptive Analysis of the Patients with an Acute Cerebrovascular Event

A total of 116 patients with AIS/TIA were included in this study. The mean age of these patients was 70±12 years; 53% of whom were male. The baseline clinical severity measured with NIHSS and Barthel Index was 4.8±6.0 and 74±30, respectively. More than half of the patients had an AIS (58%). A detailed descriptive analysis of the characteristics of the patients with acute cerebrovascular event is given in Table 1.

**Table 1:** Baseline characteristics of acute ischemic stroke (AIS)/transitory ischemic attack (TIA) patients.

Characteristic	Value (n = 116)
Age, years	70±12
Sex, n (%)	
Male	62 (53)
Female	54 (47)
Modality, n (%)	
AIS	67 (58)
TIA	49 (42)
TOAST criteria, n (%)	
Cardioembolism	70 (60)
Large-artery atherosclerosis	4 (3)
Small-vessel occlusion	12 (10)
Other determined or undetermined etiology	30 (26)
Thrombolysis, n (%)	34 (29)
Comorbidities, n (%)	
Hypertension	105 (92)
Diabetes mellitus	41 (35)
Hyperlipidemia	80 (69)
Renal failure	10 (9)
Atrial fibrillation	37 (32)
Persistent foramen ovale	28 (24)
Heart failure	5 (4)
Coronary artery disease	8 (7)
Family history of stroke	11 (10)
Smoking, n (%)	18 (16)
Platelet inhibitor before blood withdrawal, n (%)	87 (75)
Anticoagulation before blood withdrawal, n (%)	8 (7)
Lipid-lowering drug before blood withdrawal, n (%)	36 (31)
National Institutes of Health Stroke Scale at admission	4.8±6.0
Barthel Index at admission	74±30
Body mass index, kg/m^2^	27±5
HbA_1c_, mmol/mol hemoglobin	46±13
Lipid profile, mmol/L	
Total cholesterol	202±52
Low-density lipoprotein	121±45
High-density lipoprotein	51±15
Triglycerides	157±153
Duration between symptom onset and blood withdrawal, h	14±7

HbA_1c_, glycated hemoglobin; TOAST, Trial of Org 10172 in Acute Stroke Treatment.

### Comparison of VWF Levels in AIS/TIA Patients, CCD Patients, and HV

**Figure 1:**
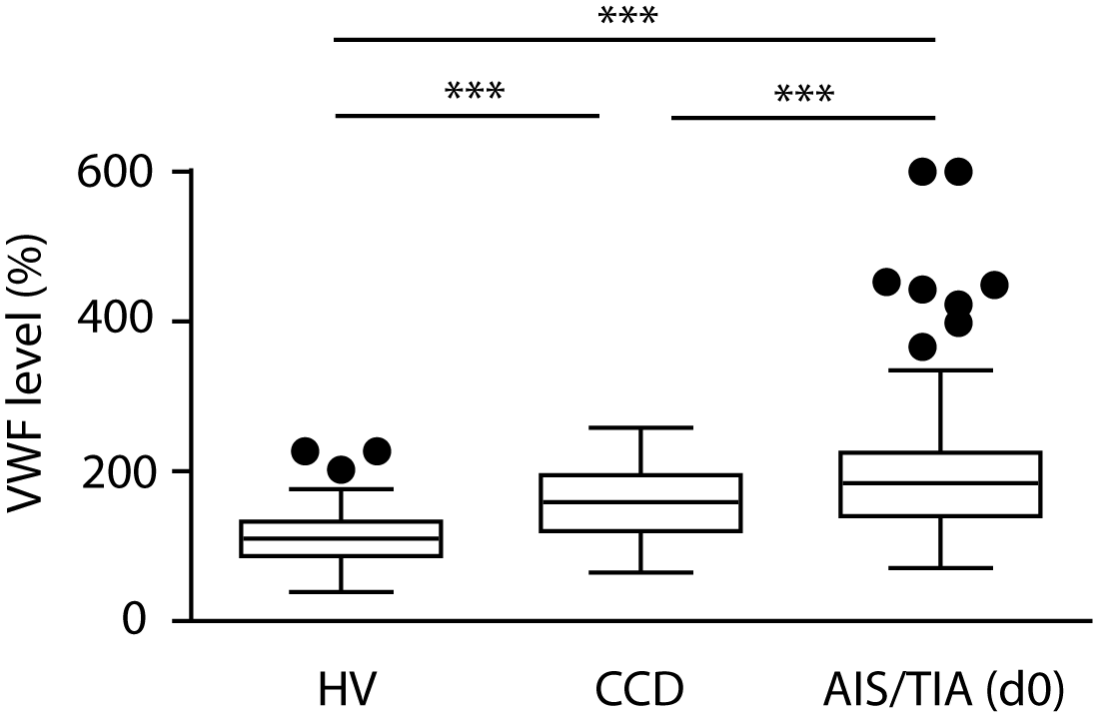
von Willebrand factor (VWF) levels in acute ischemic stroke (AIS)/transitory ischemic attack (TIA), chronic cerebrovascular disease (CCD), and healthy volunteers (HV). The VWF levels are depicted in box-and-whisker plots indicating the first and third quartiles as well as the 1.5 interquartile range (IQR, Tukey plot). Outliers outside 1.5 IQR are visualized by single dots. VWF levels showed significant differences between the three groups. Analysis of variance with Bonferroni post hoc test, ***P<0.001. d0 = day 0.

VWF levels by patient subtype with acute cerebrovascular events (AIS or TIA), or chronic cerebrovascular disorders (CCD), were compared with the levels of HV. VWF levels showed significant differences between the three groups (AIS/TIA: 200±95%; CCD: 158±46%; HV: 113±36%) (Figure 1). Both patient subtypes had significantly higher levels than HV, AIS and TIA patients even higher than CCD patients (all *P*<0.001). After adjustment for age and sex, highly significant results remained (*P*<0.001) (data not shown).

### Relationship between VWF Levels and Key Demographic and Clinical Parameters in Patients with an Acute Cerebrovascular Event

The potential relationship between VWF levels and standard laboratory parameters such as CRP or blood count was evaluated. VWF levels were significantly correlated with CRP (r = 0.30, *P* = 0.001) and different leukocyte subsets (leukocyte count: r = 0.29, *P* = 0.002; neutrophil count: r = 0.36, *P*<0.001; lymphocyte count: r = −0.28, *P* = 0.002; monocyte count: r = 0.20, *P* = 0.03) (data not shown).

The association between VWF levels and key demographic and clinical characteristics was assessed in a univariate analysis (Table 2). Age (*P* = 0.001), disease modality (TIA vs. AIS) (*P* = 0.01), and clinical severity (NIHSS: *P* = 0.001; Barthel Index: *P*<0.001) showed a significant association with serum VWF levels. VWF levels increased with patient age and clinical severity, while AIS patients had higher VWF levels than TIA patients.

**Table 2:** Predictors of von Willebrand factor (VWF) levels in acute ischemic stroke (AIS)/transitory ischemic attack (TIA) patients by univariate analysis.

	VWF (%)	*P* value
Sex, n		
Male	189±87	
Female	212±103	0.30
Age, years		
<55	157±44	
55–64	207±117	
65–74	188±76	
75–84	207±72	
>84	253±154	0.001
Disease modality		
AIS	219±101	
TIA	173±80	0.01
Modified TOAST criteria		
Cardioembolism	200±87	
Large-artery atherosclerosis	275±131	
Small-vessel occlusion	170±79	
Other determined or undetermined etiology	201±111	0.30
Duration between symptom onset and blood withdrawal, h		
<5	233±134	
5–12	184±89	
12–24	190±215	0.11
National Institutes of Health Stroke Scale		
0–4	179±71	
5–9	212±102	
10–15	220±55	
>15	299±182	0.001
Barthel Index		
0–30	286±146	
35–70	182±61	
>70	166±52	<0.001
Thrombolysis		
Yes	223±101	
No	190±91	0.09
Platelet inhibitor before blood withdrawal		
Yes	189±83	
No	218±111	0.11

TOAST = Trial of Org 10172 in Acute Stroke Treatment.

A multivariate analysis of all variables was performed using a linear regression model (Table 3). Three parameters: NIHSS>15 points, intake of platelet inhibitors before blood withdrawal, and CRP at admission, were identified as independent predictors of VWF levels. The time point of blood withdrawal (days 0, 1 and 3) did not influence VWF levels (*P* = 0.99) (data not shown).

**Table 3:** Predictors of von Willebrand factor levels in acute ischemic stroke (AIS)/transitory ischemic attack (TIA) patientsby multivariate analysis**.**

	Coefficient	95% confidence interval	*P* value
Sex			
Male	Reference		
Female Age, years	4.12±15.75	−27.13 to 35.37	0.79
<55	Reference		
55–64	37.28±27.04	−16.35 to 90.92	0.17
65–74	24.43±25.22	−25.62 to 74.47	0.34
75–84	39.70±26.68	−13.23 to 92.64	0.14
>84	37.83±33.84	−29.30 to 104.97	0.27
Disease modality	22.09±17.47	−12.58 to 56.76	0.21
National Institutes of Health Stroke Scale			
0–4	Reference		
5–9	11.20±23.52	−35.47 to 57.86	0.64
10–15	36.75±27.77	−18.35 to 91.86	0.19
>15	71.13±32.47	6.72 to 135.55	0.03
Thrombolysis	−0.68±20.35	−41.06 to 39.69	0.97
Platelet inhibitor before blood taking	−41.09±16.66	−74.13 to −8.05	0.02
C-reactive protein at admission, mg/dl	8.91±2.25	4.44 to 13.37	<0.001
Neutrophils at admission, n*1000/µl	1.03±3.85	−6.61 to 8.67	0.79

## Discussion

This study shows that VWF serum levels are significantly increased in both acute and chronic cerebrovascular disorders compared with healthy persons. Furthermore, we identified important demographic and clinical predictors of VWF levels in patients with acute IS or transitory ischemic attack.

It is known that VWF is a risk factor for coronary heart disease [21]. Despite a few studies that could not find an association between VWF levels and stroke risk or increased coagulability [22], [23], the vast majority of prospective studies point towards high VWF levels as risk factor also for AIS [12], [24]–[28]. Our findings support this hypothesis by showing that VWF levels are higher in patients with an acute cerebrovascular event or CCD than in healthy individuals. Importantly, stroke severity (NIHSS>15 points), CRP levels at admission, and the intake of platelet inhibitors in the days before blood withdrawal each independently predicted VWF levels.

Previous case–control studies demonstrated increased VWF levels in patients with IS [11], [29]–[31]. While Hanson and co-workers described that VWF levels differed between etiologic subtypes of IS, we found no such association [29]. Our finding of an association of CRP and VWF levels in the acute phase after IS highlights the role of VWF as an acute-phase reactant associated with inflammation [32] and, in particular, with thrombo-inflammation in IS [4], [33], [34]. The term “thrombo-inflammation” describes the interaction of thrombotic (e.g. coagulation factors, platelets) and inflammatory (e.g. immune cells) circuits operating at the ischemic neurovascular unit and has recently been identified as key mechanism of stroke occurrence and propagation at least in rodents [33], [34].

Elevated levels of VWF in patients with chronic cerebrovascular disease compared with healthy controls presented herein is similar to the findings of a previous study, which showed that subjects with carotid plaques have elevated VWF levels [35]. In addition, a recent report shows that atherosclerotic geometries exacerbate pathological thrombus formation post-stenosis in a VWF-dependent manner [36]. However, findings from another investigation suggest that the additional explanatory power of VWF (on top of the traditional risk factors, such as hypertension and diabetes) is comparatively low in patients with carotid atherosclerosis [37].

Growing evidence indicates that demographic and clinical parameters as well as therapeutic interventions can influence VWF levels [16], [17], but the relevance of these variables on VWF regulation in different disease settings is unclear. Our findings, and those of other researchers, underline the complexity of associations with VWF levels. On the one hand, a high VWF level at admission is indicated as a marker of severe IS; on the other hand, VWF level is influenced by a variety of other variables (e.g. ABO blood group [38]–[41], existence and composition of carotid plaques [15], [16]) which complicates its interpretation. Only recently, it has been shown that extracellular sodium levels within the high physiological range can raise VWF levels thereby increasing the risk of pathological thrombosis [42].

Our findings raise important considerations for clinical practice. It may be hypothesized that VWF level could represent a potential biomarker of stroke risk and stroke severity. Yet, the proactive screening of VWF levels on a regular basis to assess the risk of thromboembolic disease in individual patients has still not been recommended by medical societies [4]. This may be due to the lack of standardized test systems [4], or the confounding of measurements by the current lack of understanding of factors that influence VWF levels [13], [14] – which would impede the interpretation of findings and the prediction of the corresponding cerebrovascular risk.

In addition, VWF levels could perhaps provide a means to screen patients for specific treatment approaches. In various stroke models, blocking VWF by monoclonal antibodies has been shown to provide an impressive protective effect [5], [9]. Assuming that patients with high VWF levels would benefit most from such a potential future pharmacological anti-VWF therapy, it could be important to identify those patients. Based on our results, patients with severe IS without previous intake of platelet inhibitors show the highest levels of VWF and could possibly be the patient subgroup most suited to a targeted anti-VWF therapy. Of course, pharmacological targeting of VWF presents many challenges, and might be less reliable than blocking other more “inert” molecules of thrombus formation.

Our study has several limitations. Because of the case–control study design, blood withdrawal took place post-cerebrovascular event, which might lead to a reverse causation. However, the increase in VWF levels in patients with AIS persisted over 3 days. This suggests that elevated VWF levels were not due to early and transient activation of the cerebral endothelium. Rather, a pre-existing rise in VWF levels, i.e. before the occurrence of the ischemic event, might be postulated. Nevertheless, it should be emphasized that this study describes the degree and significance of associations between VWF levels and demographic/clinical parameters, but does not assign causality. To overcome this limitation, prospective clinical trials should follow this case–control study. Furthermore, for ethical reasons, we could not recruit patients who were unable to give informed consent. Thus, patients with very large strokes and/or aphasia might be under-represented in our study. Further limitations include the relatively high proportion of TIA patients (42%) for which a non-vascular origin of symptoms cannot be entirely excluded, and treatment with anticoagulants in 7% of AIS/TIA patients which might interfere with the regulation of VWF levels.

## Conclusion

In summary, we have demonstrated in this pilot study that an important potential biomarker and promising target for future therapeutic approaches, VWF, is differentially regulated in patients with acute and chronic cerebrovascular diseases. The ability to distinguish patient subtypes, or those at risk of an event, on the basis of VWF levels is a tempting approach that may enable better diagnosis or more targeted therapy in the future. In addition, this study adds to our knowledge of the factors that can influence VWF levels in a given patient. Nevertheless, prospective clinical trials are necessary to reproduce these results and to overcome the limitations that accompany retrospective study designs.
